# Expression and Immunogenicity of Two Recombinant Fusion Proteins Comprising Foot-and-Mouth Disease Virus Structural Protein VP1 and DC-SIGN-Binding Glycoproteins

**DOI:** 10.1155/2017/7658970

**Published:** 2017-10-08

**Authors:** Xinsheng Liu, Jianliang Lv, Yuzhen Fang, Peng Zhou, Yanzhen Lu, Li Pan, Zhongwang Zhang, Junwu Ma, Yongguang Zhang, Yonglu Wang

**Affiliations:** ^1^State Key Laboratory of Veterinary Etiological Biology, OIE/National Foot and Mouth Disease Reference Laboratory, Key Laboratory of Animal Virology of Ministry of Agriculture, Lanzhou Veterinary Research Institute, Chinese Academy of Agricultural Sciences, Lanzhou 730046, China; ^2^Jiangsu Co-Innovation Center for Prevention and Control of Important Animal Infectious Diseases and Zoonoses, Yangzhou 225009, China; ^3^College of Veterinary Medicine, Gansu Agricultural University, Lanzhou, Gansu 730070, China

## Abstract

Improving vaccine immunogenicity by targeting antigens to dendritic cells has recently emerged as a new design strategy in vaccine development. In this study, the VP1 gene of foot-and-mouth disease virus (FMDV) serotype A was fused with the gene encoding human immunodeficiency virus (HIV) membrane glycoprotein gp120 or C2-V3 domain of hepatitis C virus (HCV) envelope glycoprotein E2, both of which are DC-SIGN-binding glycoproteins. After codon optimization, the VP1 protein and the two recombinant VP1-gp120 and VP1-E2 fusion proteins were expressed in Sf9 insect cells using the insect cell-baculovirus expression system. Western blotting showed that the VP1 protein and two recombinant VP1-gp120 and VP1-E2 fusion proteins were correctly expressed in the Sf9 insect cells and had good reactogenicity. Guinea pigs were then immunized with the purified proteins, and the resulting humoral and cellular immune responses were analyzed. The VP1-gp120 and VP1-E2 fusion proteins induced significantly higher specific anti-FMDV antibody levels than the VP1 protein and stronger cell-mediated immune responses. This study provides a new perspective for the development of novel FMDV subunit vaccines.

## 1. Introduction

Foot-and-mouth disease (FMD) is an acute, severe, and highly contagious disease that is caused by foot-and-mouth disease virus (FMDV), which infects cloven-hoofed animals such as cattle, pigs, and sheep. FMDV is characterized by rapid transmission, high morbidity, and low mortality and can cause serious economic losses and social impacts [1, 2]. Vaccination is the most reliable and effective means of preventing and controlling FMD. Although traditional FMD vaccines play an important role in the prevention and control of FMD, they present a number of shortcomings, such as incomplete inactivation of the virus and escape of live viruses from vaccine production facilities [3, 4]. Therefore, the development of safe and effective new genetically engineered vaccines is required for the prevention, control, and eventual elimination of FMD in the future. Many genetically engineered FMDV vaccines have recently emerged, including subunit vaccines, edible vaccines, synthetic peptide vaccines, gene-deleted vaccines, live vector vaccines, and nucleic acid vaccines. However, the immune effects of these new genetically engineered vaccines are not superior to those of traditional inactivated vaccines. Therefore, vaccine research has focused on the adoption of new design strategies to further improve the immunogenicity of these new genetically engineered vaccines.

Dendritic cells (DCs) are the most potent specialized antigen-presenting cells in the body. DCs capture, process, and present antigens through their surface antigen receptors. DCs participate in the activation of naïve T-cells and induce their proliferation and differentiation to elicit a strong immune response [5, 6]. Although DCs have a potent antigen capture function, their nonspecific mechanisms of antigen capture and presentation could affect vaccine presentation and further influence the immune effects of vaccines [7]. Therefore, improvement of the immunogenicity of vaccines by targeting antigens to DCs has become an emerging new vaccine design strategy. DC-SIGN (dendritic cell-specific intercellular adhesion molecule-3-grabbing nonintegrin), also known as CD209, is a C-type lectin receptor on the surface of DC membranes that can specifically bind to a variety of ligands, including highly glycosylated proteins, Lewis-type blood antigens (Le^*x*^, Le^*y*^, Le^*a*^, and Le^*b*^), intercellular adhesion molecules, and some virus envelope glycoproteins [8, 9]. The use of chemical crosslinking or genetic engineering methods to combine vaccine antigens with the DC-SIGN ligand can specifically target vaccine antigens to DCs and thus significantly improve the immune effects of a vaccine [8, 10–13].

The FMDV capsid protein consists of four structural proteins (VP1, VP2, VP3, and VP4). The VP1 protein is exposed on the surface of the viral capsid and is the main protein that determines the serotype and genotype of FMDV. The VP1 protein contains several antigen epitopes that can stimulate the production of effective humoral and cellular immune responses and induce the production of specific neutralizing antibodies [1]. Related studies have shown that the HIV membrane glycoprotein gp120 and the HCV envelope glycoprotein E2 are both high-affinity ligands for DC-SIGN that are capable of specifically binding to DC-SIGN to mediate the internalization and presentation of exogenous antigens [14–18]. In this study, the DC-SIGN-specific ligands HIV membrane glycoprotein gp120 and HCV envelope glycoprotein E2 were separately fused with the FMDV structural protein VP1 and expressed using the insect cell-baculovirus expression system. The immunogenicity of these two DC-targeting recombinant FMDV VP1 fusion proteins was evaluated in guinea pigs and compared with that of the VP1 protein.

## 2. Materials and Methods

### 2.1. Ethics Statement

All guinea pig experiments were performed in a biosafety level 3 laboratory at Lanzhou Veterinary Research Institute (LVRI), Chinese Academy of Agricultural Sciences (CAAS). This study was performed with the approval of the Institutional Animal Use and Care Committee of CAAS, and all experiments were performed according to local (Regulations for the Administration of Affairs concerning Experimental Animals) and international (Dolan K. 2007 Second Edition of Laboratory Animal Law; Blackwell, UK) guidelines on the ethical use of animals. All guinea pigs used in the present study were humanely bred during the experiment and euthanized at the end of the experiment.

### 2.2. Virus, Plasmids, Cells, and Reagents

FMDV serotype A strain AF/72 was isolated and maintained in our laboratory (the strain was maintained and provided by the Lanzhou Veterinary Research Institute (LVRI), Chinese Academy of Agricultural Sciences (CAAS)).* E. coli* TOP10 competent cells, the baculovirus transfer vector pFastBac 1, and* E. coli *DH10Bac competent cells were purchased from Invitrogen (California, USA).* Spodoptera frugiperda *(Sf9) insect cells (Invitrogen, USA) were cultured in Sf-900™ II SFM medium (Invitrogen, USA) containing 5% heat-inactivated fetal bovine serum (FBS; Gibco, USA) at 27°C in an incubator with 5% CO_2_. Restriction enzymes were purchased from New England Biolabs (NEB); the transfection Cellfectin® II Reagent and Grace's Insect Medium were purchased from Invitrogen; the mouse anti-His tag monoclonal antibody and HRP-conjugated goat anti-mouse IgG were purchased from Abbkine (California, USA).

### 2.3. Sequence Design and Synthesis

The RNeasy Mini Kit (Qiagen, USA) was used to extract genomic RNA from the FMDV serotype A strain AF/72 stored in our laboratory. The sequence of the gene encoding the structural protein VP1 was amplified using the primer pair VP1-F/R (VP1-F: 5′-ACTACCACCACTGGTGAG-3′ and VP1-R: 5′-TTACAGCAGCTGCTTGGCAGG-3′). The PCR product was cloned using the TOPO® TA Cloning® Kit (Invitrogen, USA), and the resulting plasmids were sent to Sangon Biotech (Shanghai, China) for sequencing. The VP1 gene sequence of the AF/72 strain obtained from sequencing was optimized according to the codon preference of the insect cells by Sangon Biotech (Shanghai, China), along with introduction of a His tag at the end of the VP1 sequence and the restriction enzyme sites* Bam*HI and* Hin*dIII. The sequence of the C2-V3 region of HIV glycoprotein gp120 (accession number CAA74763) or the HCV envelope glycoprotein E2 (accession number JN870282) published in GenBank was fused with the VP1 sequence of FMDV serotype A strain AF/72 through a (Gly_3_Ser)_5_ linker to obtain two recombinant fusion genes with codon preferences consistent with that of insect cells. Similarly, the restriction enzyme sites* Bam*HI and* Hin*dIII and a His tag were introduced in the fusion sequence and synthesized by Sangon Biotech (Shanghai, China).

### 2.4. Construction of Recombinant Baculovirus Plasmids

The synthesized gene products encoding VP1, VP1-gp120, VP1-E2, and the pFastBac 1 vector (Invitrogen, USA) were digested with* Bam*HI and* Hin*dIII. Then, the extracted VP1, VP1-gp120, and VP1-E2 fragments were ligated separately with the pFastBac 1 fragment using T4 DNA ligase at 16°C for 10 hours. The ligation products were transformed into* E. coli *DH5*α* competent cells, and the positive clones obtained by blue-white screening were inoculated into LB medium containing ampicillin and cultured at 37°C for 12 hours in an incubator shaker at 220 rpm. The plasmids pFastBac-VP1, pFastBac-VP1-gp120, and pFastBac-VP1-E2 were extracted from the positive clones and confirmed by restriction enzyme digestion. The constructed recombinant transfer plasmids pFastBac-VP1, pFastBac-VP1-gp120, and pFastBac-VP1-E-2 were used to transform* E. coli* DH10Bac competent cells. Positive colonies were selected by blue-white screening, and the recombinant bacmids were extracted and characterized using PCR with the universal M13 primers (M13-F: 5′-GTTTTCCCAGTCACGAC-3′ and M13-R: 5′-CAGGAAACAGCTATGAC-3′). The correct recombinant bacmids were named rBacmid-VP1, rBacmid-VP1-gp120, and rBacmid-VP1-E-2, respectively.

### 2.5. Preparation of Recombinant Baculoviruses

A 1 *μ*g quantity of the recombinant bacmids and 6 *μ*L of Cellfectin II Reagent were separately diluted using 100 *μ*L of incomplete Grace's medium (without antibiotics and FBS). After mixing well, the two mixtures were combined, gently mixed, and then incubated at room temperature for 45 minutes to prepare the rBacmid liposomes. Sf9 monolayer cells that were in a good growth stage in 6-well plates (9 × 10^5^ cells/well) were washed twice with incomplete Grace's medium and then covered with the rBacmid liposomes. The cells were incubated at 27°C for 5 hours. The culture medium was discarded, complete Grace's medium (containing antibiotics and FBS) was added, and the cells were incubated at 27°C. After the appearance of cytopathic effects, the cells were removed from the plate, placed in a centrifuge tube, shaken vigorously, and centrifuged at 1000 ×g for 15 minutes. The supernatants containing the P1 recombinant baculoviral stocks (rBac-VP1, rBac-VP1-gp120, and rBac-VP1-E2) were collected. The recombinant baculovirus was subcultured in Sf9 cells to the second passage (titer of approximately 10^7^ pfu/mL) and then stored at 4°C for later use.

### 2.6. Expression and Purification of Recombinant Proteins

Sf9 cells were seeded into 1000 mL cell culture flasks at a density of 2 × 10^6^ cells/mL and infected with the P2 recombinant baculovirus rBac-VP1, rBac-VP1-gp120, or rBac-VP1-E2 when the cells were in the logarithmic phase. After culturing for 48–72 hours, the cells and culture medium were transferred to centrifuge tubes and centrifuged at 10,000*g* for 20 minutes at 4°C. The cells were collected and lysed after the addition of protease inhibitor (1 : 100) by pulse sonication of 6 seconds at 250 W at 3-second intervals for a total of 4 minutes. The cell lysate was centrifuged at 10,000*g* for 10 minutes at 4°C. The supernatant was collected and passed through a Ni-chelating affinity column at a flow rate of 0.5 mL/minute. The Ni column was equilibrated with 20 mM PB buffer at a flow rate of 0.5 mL/minute until the OD280 of the effluent reached baseline. The column was washed with Ni-IDA Washing Buffer (20 mM PB, 30 mM imidazole, and 0.15 M NaCl, pH 8.0) at a flow rate of 1 mL/min until the OD280 of the effluent reached baseline. Then, the target protein was eluted with Ni-IDA Elution Buffer (20 mM PB, 300 mM imidazole, and 0.15 M NaCl, pH 8.0) at a flow rate of 1 mL/min, and the effluent was collected. The collected recombinant protein solution was added to a dialysis bag, dialyzed against 1x PBS overnight, and then subjected to 10% sodium dodecyl sulfate polyacrylamide gel electrophoresis (SDS-PAGE).

### 2.7. Western Blotting

The purified proteins were separated by SDS-PAGE, and the proteins in the gel were then transferred to a membrane under a constant voltage of 100 V for 1.5 hours. Following the completion of the transfer, the membrane was washed with PBS 4 times for 5 minutes per wash. The membrane was blocked with 5% skimmed milk at 37°C for 1 hour, followed by incubation with the primary mouse anti-His-tag monoclonal antibody (1 : 1000 dilution) and type A FMDV VP1 monoclonal antibody (prepared and stored in our laboratory, 1 : 1000 dilution) for 1 hour at 37°C, respectively. The membrane was washed with TBS-Tween (50 mM Tris, 150 mM NaCl, and 0.05% Tween 20, pH 7.6) 4 times for 5 minutes per wash, followed by incubation with the secondary horseradish peroxidase- (HRP-) labeled goat anti-rabbit IgG antibody (Sigma, USA) at a 1 : 5000 dilution for 1 hour at 37°C. The membrane was washed and visualized using an ECL chemiluminescent substrate reagent kit (Thermo Scientific, USA).

### 2.8. Guinea Pig Immunization

All animal procedures were approved by the Ethics Committee of LZVR. Twenty-five specific pathogen-free- (SPF-) grade healthy guinea pigs weighing 250–300 g were randomly divided into 5 groups with 5 animals per group. The experimental groups* (three groups, each group containing 5 guinea pigs)* were immunized by intramuscular injection of 1 mL of purified VP1 protein or the VP1-gp120 or VP1-E2 fusion proteins containing approximately 0.2 mg purified proteins with ISA-206 adjuvant (China Agricultural Vet. Bio. Science and Technology Co., Lanzhou, China) at an adjuvant : antigen ratio of 1 : 1 on days 0 and 21, respectively. A PBS/ISA 206 mixture was used as the negative immunization control (*n* = 5), and the commercially available inactivated vaccine (China Agricultural Vet. Bio. Science and Technology Co., Lanzhou, China) was used as the positive immunization control (*n* = 5). Blood samples were collected on days 7, 14, 21, and 28 after the first immunization, and serum was isolated for antibody and cytokine detection.

### 2.9. Detection of Anti-FMDV-Specific Antibodies by ELISA

The FMDV-specific IgG antibody titers were measured in the collected guinea pig serum samples via an indirect enzyme-linked immunosorbent assay (ELISA). In detail, the inactivated whole-virus antigen of FMDV serotype A (Diagnostic Products Center, LVRI, Lanzhou, China) was diluted with 0.1 M bicarbonate buffer (pH 9.6) at a 1 : 5 ratio. Then, a 96-well flat-bottomed plate was coated with the diluted antigen at 100 *μ*L per well. The plate was sealed and incubated at 4°C overnight. The plate was washed with PBST 3 times and blocked with 5% skimmed milk in PBST for 1 hour at 37°C. The plate was then washed with PBST 3 times, followed by the addition of 100 *μ*L of the serum samples (1 : 10 dilution) and incubation at 37°C for 1 hour. Control wells were set aside for the negative, positive, and blank controls. The plate was washed with PBST 3 times, followed by incubation with 100 *μ*L of HRP-labeled rabbit anti-guinea pig IgG diluted at 1 : 1000 (Diagnostic Products Center, LVRI, Lanzhou, China) for 1 hour. The plate was washed with PBST 5 times, followed by the addition of 50 *μ*L of the* o*-phenylenediamine dihydrochloride (OPD) substrate and incubation at 37°C for 15 minutes. Then, 50 *μ*L of stop solution was added, and absorbance was determined at 492 nm.

### 2.10. Virus Neutralizing Antibody Test (VNT)

The FMDV-specific neutralizing antibody titers from serum samples at 7 days after the booster immunization were determined using the VNT with BHK-21 cells according to the OIE protocol [19]. Briefly, 50 *μ*L of twofold serial serum dilutions starting at 1 : 2 was coincubated with equal volumes of viral stock containing 100 TCID_50_ (50% tissue culture infective doses) of FMDV AF/72 in 96-well plates (Corning, USA) at 37°C for 1 h. Then, cells were added to the mixture as indicators of residual infectivity. The plates were incubated at 37°C for 72 h, and the cells were fixed and stained with 10% methanol and 0.05% methylene blue solution (prepared with formaldehyde solution). The neutralizing antibody titers were evaluated as the reciprocal log_10_ of the highest dilution that neutralized 100 TCID_50_ of FMDV in 50% of the wells.

### 2.11. Lymphocyte Proliferation Assay

Blood samples were collected from the guinea pigs in each group at 7 days after the booster immunization. Peripheral blood lymphocytes were isolated using the Guinea Pig Lymphocyte Separation Solution Kit (Solarbio, China). The isolated lymphocytes were resuspended in RPMI 1640 medium containing 1% antibiotics, seeded into a 96-well plate at a density of 1 × 10^5^ cells/well, and cultured in a 5% CO_2_ incubator at 37°C for 24 hours to allow attachment of the cells to the plate. Then, the FMDV antigen was added to each sample at a final concentration of 10 *μ*g/mL. For each sample, RPMI 1640 medium was added as a negative control, and concanavalin A (ConA) at a final concentration of 5 *μ*g/mL was added as the positive control. After incubation at 37°C for 72 hours, cell proliferation was examined using the MTT Cell Proliferation Assay Kit (Solarbio, China). In detail, the supernatant was carefully removed, and 90 *μ*L of fresh medium was added, followed by the addition of 10 *μ*L of MMT and incubation for 4 hours. After incubation, the supernatant was discarded, and 110 *μ*L of formazan solubilization solution was added. The plate was placed on a shaker at low speed for 10 minutes to fully dissolve the crystals. The OD value of each well was read at 490 nm. The results were expressed as the stimulation index (SI, ratio of stimulated sample : unstimulated sample at OD490 nm).

### 2.12. Cytokine Analysis

Serum samples were collected from the guinea pigs in each group at 7 days after booster immunization. The levels of the cytokine IFN-*γ* were examined in the serum samples from each group using the Rat Cytokine Antibody Array CYT-1 (RayBiotech, Norcross, GA, USA), and differences in the IFN-*γ* levels were analyzed. The fluorescence signals were read using an InnoScan 300 Microarray Scanner and analyzed using the data analysis software QAM-CYT-1. The assay and data analysis were performed by the RayBiotech Technical Service Department (Guangzhou, China).

### 2.13. Statistical Analysis

Statistical significance among the different experimental groups was determined using the one-way ANOVA. Differences were considered significant when the *P* value was less than 0.05.

## 3. Results

### 3.1. Confirmation of the Recombinant Transfer Vectors and Bacmids

**Figure 1:**
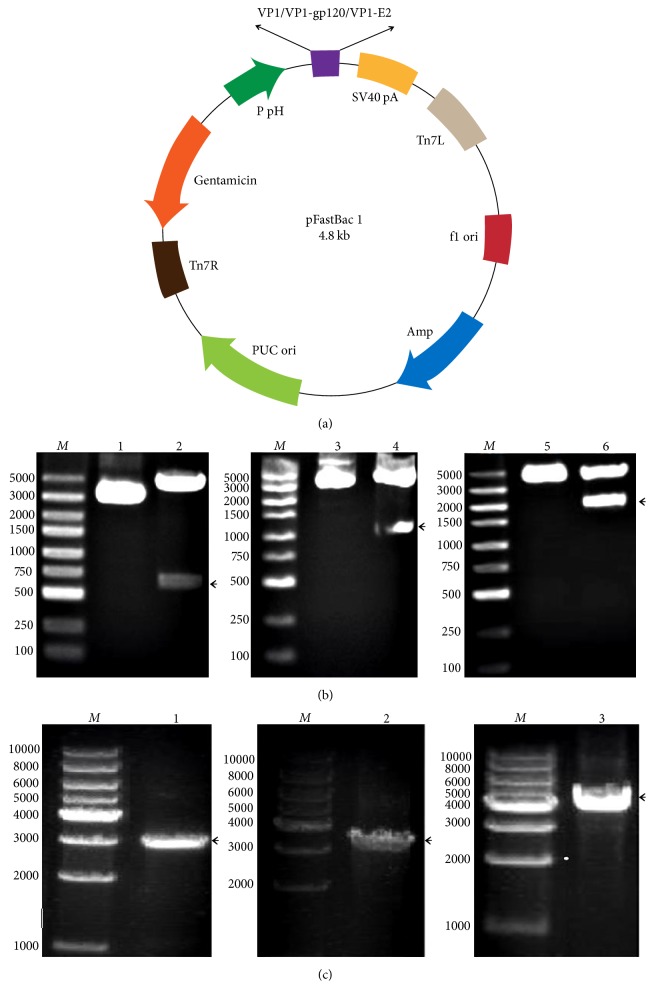
Design of the recombinant transfer vector for the VP1 protein and the VP1-gp120 and VP1-E2 fusion proteins and confirmation of the recombinant transfer vector and baculovirus bacmids. *M* indicates the DNA size marker. (a) Sites of insertion of the coding sequences for the VP1 protein and the VP1-gp120 and VP1-E2 fusion proteins in the baculovirus transfer vector pFastBac 1. (b) The recombinant transfer vectors pFastBac-VP1, pFastBac-VP1-gp120, and pFastBac-VP1-E2 were digested with the restriction endonucleases* Bam*HI and* Hin*dIII and subjected to agarose gel electrophoresis. Lanes 1, 3, and 5: undigested plasmids. Lanes 2, 4, and 6: pFastBac-VP1, pFastBac-VP1-gp120, and pFastBac-VP1-E2 digested with* Bam*HI and* Hin*dIII, respectively. (c) The recombinant bacmids rBacmid-VP1, rBacmid-VP1-gp120, and rBacmid-VP1-E2 were amplified by PCR with the M13 universal primers, and the PCR products were subjected to agarose gel electrophoresis. Lanes 1, 2, and 3: PCR products from rBacmid-VP1, rBacmid-VP1-gp120, and rBacmid-VP1-E2, respectively.

The synthesized gene products for the VP1 proteins and the VP1-gp120 and VP1-E2 recombinant fusion proteins were digested at the* Bam*HI and* Hin*dIII recognition sites and cloned into the pFastBac 1 vector to construct the recombinant transfer vectors pFastBac-VP1, pFastBac-VP1-gp120, and pFastBac-VP1-E2, respectively (Figure 1(a)). The recombinant transfer vectors were digested with* Bam*HI and* Hin*dIII to obtain fragments of 675 bp, 1073 bp, and 1944 bp, respectively, by agarose gel electrophoresis; the size of each fragment was consistent with the expected size (Figure 1(b)). The sequencing results indicated that the VP1, VP1-gp120, and VP1-E2 sequences in the recombinant transfer vectors were correct. Additionally, the recombinant bacmids rBacmid-VP1, rBacmid-VP1-gp120, and rBacmid-VP1-E2 were confirmed by PCR with the M13 universal primers to yield specific amplification fragments of approximately 2.9 kb, 3.3 kb, and 4.2 kb, respectively (Figure 1(c)), indicating that the recombinant bacmids were constructed correctly.

### 3.2. Expression and Characterization of the Recombinant Proteins

**Figure 2:**
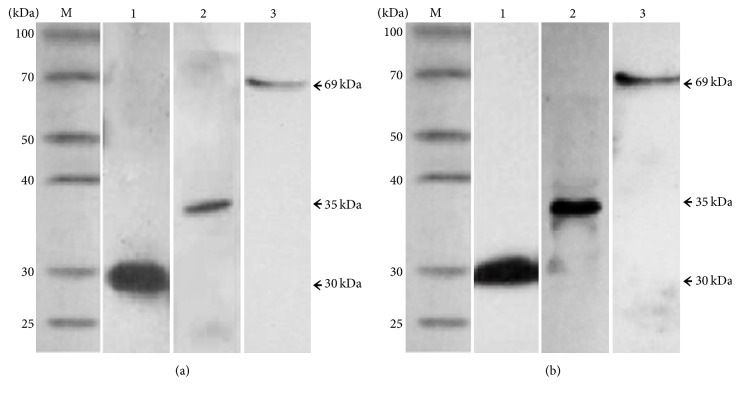
Western blotting analysis of the VP1 protein and the VP1-gp120 and VP1-E2 fusion proteins. (a) Western blotting analysis using the mouse anti-His tag monoclonal antibody as the primary antibody. Lanes 1, 2, and 3 correspond to the VP1 protein and the VP1-gp120 and VP1-E2 fusion proteins, respectively. (b) Western blotting analysis using the anti-type A FMDV VP1 monoclonal antibody as the primary antibody. Lanes 1, 2, and 3 correspond to the VP1 protein and the VP1-gp120 and VP1-E2 fusion proteins, respectively.

Apparent cytopathic effects were observed in the Sf9 insect cells 72 hours after transfection with the recombinant baculoviral bacmids rBacmid-VP1, rBacmid-VP1-gp120, and rBacmid-VP1-E2. These cytopathic effects manifested as enlarged round cells with enlarged nuclei that filled the entire cytoplasm and poorly refractive particles in the nuclei. The cells and supernatants were harvested and lysed, and the expressed proteins were detected by western blotting. The results showed that the VP1 protein and the VP1-gp120 and VP1-E2 recombinant fusion proteins were expressed correctly in the Sf9 cells; the molecular weights of the expressed proteins were approximately 30 kDa, 35 kDa, and 69 kDa, respectively (Figure 2). Additionally, the expressed proteins reacted not only with the mouse anti-His tag monoclonal antibody (Figure 2(a)) but also with the anti-FMDV VP1 monoclonal antibody (Figure 2(b)), indicating that the expressed VP1 protein and the VP1-gp120 and VP1-E2 recombinant fusion proteins had good reactogenicity.

### 3.3. Antibody Responses during Immunization

**Figure 3:**
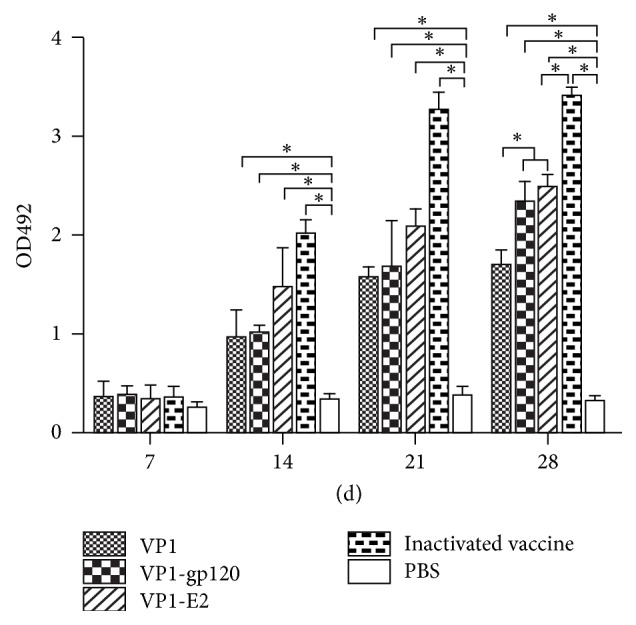
The VP1 protein and the VP1-gp120 and VP1-E2 fusion proteins induced the production of specific anti-FMDV IgG antibodies in guinea pig serum* (each group contained 5 guinea pigs, n* = 5). Serum samples were collected from all guinea pigs at 7 days after booster immunization, and the specific anti-FMDV IgG antibody levels were determined by indirect ELISA. PBS/ISA 206 mixture was used as the negative control (*n* = 5), and the commercially available inactivated vaccine was used as the positive control (*n* = 5).* Significant values *(^*∗*^*P* < 0.05)* are indicated by an asterisk*.

To assess the immunogenicity of the VP1 protein and the VP1-gp120 and VP1-E2 recombinant fusion proteins in guinea pigs, blood samples were collected from all guinea pigs at 7 days after booster immunization, and anti-FMDV-specific IgG levels in the serum were determined using indirect ELISA. As shown in Figure 3, the VP1 protein, the VP1-gp120 and VP1-E2 recombinant fusion proteins, and the inactivated vaccine all effectively induced specific anti-FMDV serotype A IgG antibodies, and significant increases in serum IgG antibody levels were observed over time after the initial immunization in all groups except the PBS negative control group. The serum IgG antibody levels induced by the VP1 protein and the VP1-gp120 and VP1-E2 recombinant fusion proteins were significantly lower than the levels produced in the traditional inactivated vaccine group (*P* < 0.05, Figure 3) but significantly higher than the levels in the PBS group (*P* < 0.05, Figure 3) within 28 days after the first immunization. Importantly, the serum IgG antibody levels induced by the VP1-gp120 and VP1-E2 recombinant fusion proteins were higher than the levels induced by the VP1 protein alone (*P* < 0.05, Figure 3) after booster immunization, suggesting that fusion of the VP1 protein to the gp120 and E2 proteins enhanced the ability of the VP1 protein to induce specific anti-FMDV IgG antibodies in guinea pigs.

**Figure 4:**
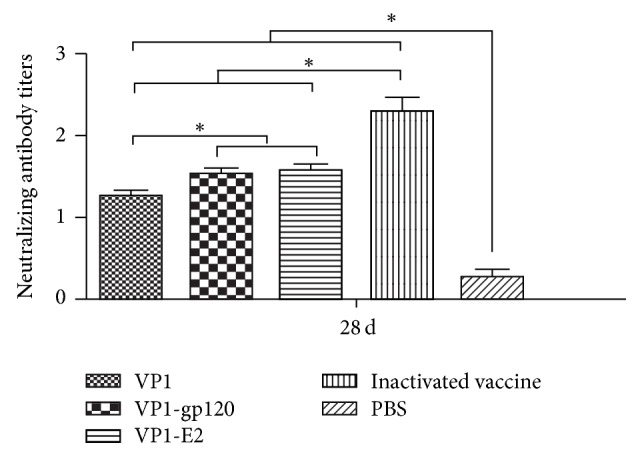
Serum neutralizing antibody titers of immunized guinea pigs* (each group contained 5 guinea pigs, n* = 5) at 7 days after booster immunization.* Significant values *(^*∗*^*P* < 0.05)* are indicated by an asterisk*.

Furthermore, the specific neutralizing antibodies against FMDV serotype A of the immunized guinea pigs in each group at 7 days after the booster immunization were assessed by VNT (Figure 4). The neutralizing antibody titers in the VP1 protein, the VP1-gp120 and VP1-E2 recombinant fusion proteins, and the inactivated vaccine groups showed significantly higher FMDV-neutralizing activity than the antibody responses of the PBS group (*P* < 0.05, Figure 4). Meanwhile, the neutralizing antibody titers of VP1-gp120 and VP1-E2 recombinant fusion proteins were higher than the titers induced by the VP1 protein (*P* < 0.05, Figure 4), indicating that recombinant fusion strategy could enhance the ability of the VP1 protein to induce specific neutralizing antibody in guinea pigs.

### 3.4. Cell-Mediated Immune Responses

**Figure 5:**
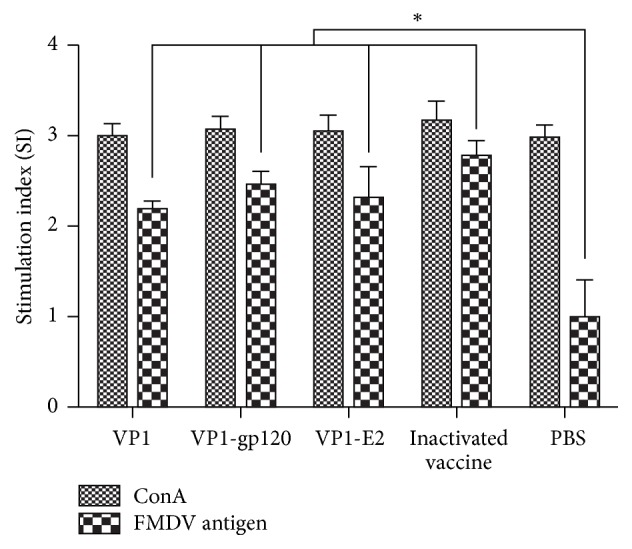
Specific proliferative responses of peripheral blood lymphocytes from guinea pigs detected by MTT colorimetry.* Stimulation index (SI) means the ratio of stimulated sample : unstimulated sample at OD490 nm*.* Significant values *(^*∗*^*P* < 0.05)* are indicated by an asterisk*.

**Figure 6:**
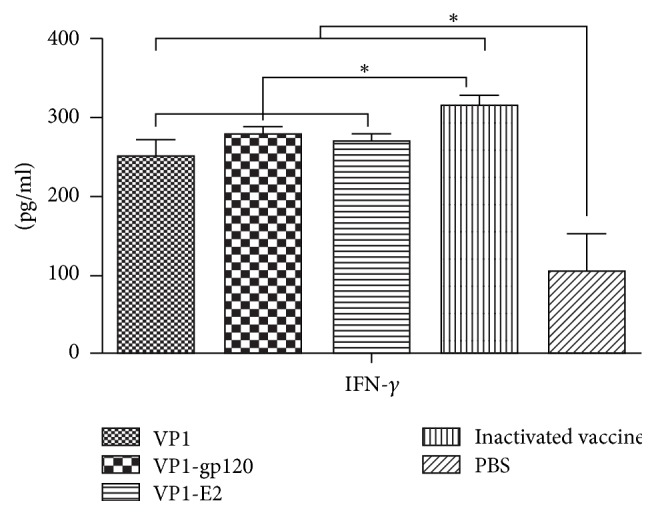
IFN-*γ* levels induced by the VP1 protein (*n* = 5) and the VP1-gp120 (*n* = 5) and VP1-E2 (*n* = 5) fusion proteins in guinea pig serum.* Significant values *(^*∗*^*P* < 0.05)* are indicated by an asterisk*.

Peripheral blood lymphocytes were isolated from the guinea pigs 7 days after booster immunization. The specific proliferative responses of the peripheral lymphocytes from the guinea pigs were examined using the MTT colorimetric assay. The proliferative responses in splenic lymphocytes from all guinea pigs were stimulated by concanavalin A (Figure 5), with no significant differences between groups (*P* > 0.05, Figure 5). Differences in lymphocyte proliferation were observed among all groups incubated with the inactivated FDMV antigen. In detail, lymphocytes from guinea pigs immunized with the VP1 protein, VP1-gp120 and VP1-E2 recombinant fusion proteins, or inactivated vaccine exhibited significantly higher proliferation levels than the PBS control group (*P* < 0.05, Figure 5). The lymphocytes of the group immunized with the inactivated vaccine showed the highest proliferation level, followed by the groups immunized with the recombinant proteins in the order VP1-gp120, VP1-E2, and VP1 protein. Although lymphocyte proliferation was higher in the VP1-gp120 and VP1-E2 immunization groups compared to the VP1 protein immunization group, the differences between the groups were not significant (*P* > 0.05, Figure 5). Furthermore, to assess the cytokine levels in guinea pig sera after immunization, the serum IFN-*γ* content was measured using a commercially available cytokine antibody array. The results showed that the VP1 protein, the VP1-gp120 and VP1-E2 recombinant fusion proteins, and the inactivated vaccine induced higher IFN-*γ* levels in guinea pigs than in the PBS negative control (*P* > 0.05, Figure 6). The ability of the inactivated vaccine to induce IFN-*γ* production was significantly greater than the ability of the three recombinant proteins (*P* > 0.05, Figure 6), whereas no significant differences (*P* < 0.05, Figure 6) were observed between the VP1, VP1-gp120, and VP1-E-2 recombinant proteins.

## 4. Discussion

The baculovirus insect cell expression system is one of the most commonly used eukaryotic expression systems and is fast and efficient. Hundreds of genes from animals, plants, viruses, bacteria, and fungi have been highly expressed in insect cells [20–22]. The expressed exogenous proteins can be posttranslationally modified in the cell (i.e., glycosylation, phosphorylation, and acylation), and therefore this expression system can be used to obtain a large number of soluble recombinant proteins that are functionally similar to the natural proteins [20, 23]. The HIV gp120 and HCV E2 proteins are both viral envelope glycoproteins that play important roles in mediating binding of the virus to its receptor. The glycosylation of these proteins has a great impact on their antigenicity. Eukaryotic expression systems can glycosylate the expressed proteins and maintain their natural structure to the maximum extent. Therefore, this study used the baculovirus insect cell eukaryotic expression system to express the VP1 protein and the VP1-gp120 and VP1-E2 recombinant fusion proteins.

Protein subunit vaccines contain only viral capsid proteins without nucleic acids and therefore are very safe. These vaccine types have become one of the main research directions in research on FMDV vaccines [24]. The VP1 protein is the main antigenic protein in FMDV and can induce the production of protective neutralizing antibodies to provide a greater protective effect. Therefore, many studies of FMDV subunit vaccines have focused on the VP1 protein [25–29]. However, FMDV subunit vaccines have lower immunological effects than traditional inactivated vaccines [24]. Therefore, targeting vaccines to dendritic cells to improve their immune effects has become an important strategy to improve the immune effects of vaccines. DC-SIGN is the main ligand used for DC targeting [10]. Therefore, in this study, natural ligands (the gp120 and E2 proteins) with a high affinity for DC-SIGN were selected as the targeting molecules to enhance the efficiency of targeted presentation of the VP1 protein to DCs through the expression of a VP1 fusion protein with these two DC-SIGN ligands; this approach was intended to improve the immune effect of the VP1 protein as a subunit vaccine in animals.

In gene expression studies, much attention has been paid to the selection of appropriate expression vectors and host systems, whereas the issue of whether the gene itself is the best match with the vector and host system is often overlooked. In fact, each organism used for protein expression, including* E. coli*, yeast, mammalian cells, plant cells, and insect cells, exhibits some degree of codon usage bias or preference [30, 31]. Thus, the codons of the gene sequences to be expressed greatly determine their expression efficiency in a given host [32]. Generally, rare codons can be eliminated in protein expression by redesigning and synthesizing the gene with optimal codons to achieve optimal expression of the target gene [32]. Therefore, the gene sequences encoding the VP1 protein and the VP1-gp120 and VP1-E2 recombinant fusion proteins were optimized based on the insect cell codon preference to enable efficient and accurate expression of the target proteins in Sf9 insect cells.

Western blotting analysis showed that the recombinant proteins not only reacted with the mouse anti-His-tag monoclonal antibody but also bound to the anti-type A FMDV VP1 monoclonal antibody, indicating good reactivity of the expressed recombinant proteins. Moreover, the His tag in the expression vector facilitated subsequent protein purification.

The VP1-gp120 and VP1-E2 recombinant fusion proteins induced higher anti-FMDV IgG antibody titers compared to expression of the VP1 protein alone (*P* < 0.05, Figure 3), suggesting that fusion of the VP1 protein with the gp120 and E2 proteins enhanced the ability of the VP1 protein to induce specific anti-FMDV IgG antibody production in guinea pigs. However, no significant differences in cellular immunity were found between the VP1 protein and the VP1-gp120 and VP1-E2 fusion proteins (*P* > 0.05, Figure 5). Thus, further study is necessary to clarify whether the higher anti-FMDV IgG antibody titers induced by the VP1-gp120 and VP1-E2 recombinant fusion proteins were a result of enhanced specific DC targeting of the VP1 protein through the expression of the VP1 fusion protein with the DC-SIGN ligand gp120 and E2 proteins.

## 5. Conclusion

In this study, the Bac-to-Bac baculovirus expression system was employed to successfully express the type A FMDV structural protein VP1 and its recombinant fusion proteins VP1-gp120 and VP1-E2 in insect cells, and the expressed proteins showed good reactivity. The immunization study results in guinea pigs showed that the VP1 protein and the VP1-gp120 and VP1-E2 recombinant fusion proteins induced the production of specific anti-FMDV IgG antibodies in guinea pigs, with the VP1-gp120 and VP1-E2 recombinant fusion proteins exhibiting a stronger ability to induce specific anti-FMDV IgG antibodies than expression of the VP1 protein alone. Additionally, the VP1 protein and the VP1-gp120 and VP1-E2 recombinant fusion proteins stimulated the specific proliferation of guinea pig lymphocytes and induced increased IFN-*γ* levels. In summary, the immunogenicity of antigens can be improved to a certain extent through fusion of the antigens with DC-targeting ligands, which provides a new perspective for future studies of subunit vaccines.
